# “For Example” Formulations and the Interactional Work of Exemplification

**DOI:** 10.1007/s10746-023-09665-7

**Published:** 2023-03-01

**Authors:** Yeji Lee, Jakub Mlynář

**Affiliations:** 1 University of Basel, Basel, Switzerland; 2 HES-SO Valais-Wallis University of Applied Sciences and Arts Western Switzerland, Sierre, Switzerland

**Keywords:** Classroom discourse, Conversation analysis, Ethnomethodology, Exemplification, Social interaction

## Abstract

Members in society make ubiquitous use of examples as a resource to engage in their everyday and specialized activities. This paper takes the resourcefulness of exemplification as a topic of inquiry by focusing on the formulative phrase “for example,” investigating its interactional work within the analytic framework of ethnomethodology and conversation analysis. The data used consists of 11 h of video-recordings of English as a Foreign Language classroom lessons over a semester. We conceptualize exemplification as a holistic configuration (*gestalt*) where its work consists in the production and recognition of a pair, namely the *exemplifying component* and the *exemplified component*. We demonstrate how the teacher and students position the formulative phrase as a recognizable practice for the organization of two distinct actions: *accounting for one’s opinion* and *confirming an understanding*. Our findings also present the different forms of exemplification, including elaborate narrative constructions, single terms or phrases, and specimen performances.

## Introduction

Exemplification as an interactional resource, in all its forms, constitutes an indispensable part of how we make sense of each other in everyday life.^1^ It is through the felicitous use of examples here and there in our talk-in-interaction that we are able to maintain common understanding. In the course of our interactions, examples are given and understood as recognizable entities by which we are able to not only make sense of what is, and is not, an example given by the other, but also request, at the right time, for the right reasons, an example for various purposes. Exemplification, in this sense, is reflexively achieved and accounted for as a “social fact” (Durkheim, 1895/1982; Garfinkel, 1988) in and through social interaction.

Exemplification, in and as its work, rests in formulating the relationship between the *exemplifying* component (i.e., the “example given”) and the *exemplified* component (i.e., the “target concept”). In order to adequately grasp any example, one has to know what the example is an example of, since it is only within the configuration of the pair—exemplifying and exemplified—that the two ingredients are intelligible by reference to each other. When this relationship is unclear, exemplification becomes obscure, as we can aptly illustrate with Gregory Bateson’s recollection of his teaching experience at the Veterans Administration Hospital in Palo Alto:

Gradually I discovered that what made it difficult to tell the class what the course was about was the fact that my way of thinking was different from theirs. . . . At the end of the session, one resident came up. He glanced over his shoulder to be sure that the others were all leaving, and then he said rather hesitantly. “I want to ask a question.” “Yes.” “It’s—do you want us to learn what you are telling us?” I hesitated a moment, but he rushed on with, “Or is it all a sort of example, an illustration of something else?” “Yes, indeed!”

But an example of what?

And then there was, almost every year, a vague complaint which usually came to me as a rumor. It was alleged that “Bateson knows something which he does not tell you,” or “There’s something behind what Bateson says, but he never says what it is.”

Evidently I was not answering the question, “An example of what?”

(Bateson, 1972: xvii–xviii)

While examples can be found in every corner of our social worlds, one of the more perspicuous settings (Garfinkel, 2002: 181f.) for examining its interactional work may be the classroom, where new concepts are often instructed and mediated through known examples. A case in point is the language-learning classroom, where the interactional work of examples may go beyond exemplifying grammatical rules but also manages communication more generally, not to mention other interactional concerns. There has, however, up to now been scant research conducted to date on examples’ work in the classroom from an interactional perspective (but see Lee, 2004; Oliveira et al., 2020; Wortham, 1994). The preliminary finding that examples and their work have generally been overlooked as topics in the social sciences furnishes the motivation for our thorough exploration of exemplification via sequential analysis of video-recorded, naturally occurring courses of action.

We find that examples are given by members in the classroom in a variety of ways in which they are not always explicitly named as such in the ongoing interaction. This then begs the question of why they are sometimes explicitly named, or “formulated” (Garfinkel & Sacks, 1970), as examples, such as through the phrase “for example”. How does such a phrase and its sequential design in talk-in-interaction contribute to composing for itself the configuration of a pair, or a *gestalt,* of exemplification as an intelligible social practice? The current study addresses these issues by using the formulative phrase “for example” as a unique analytic entry point to launch an investigation of the larger phenomenon of exemplification. Examining all instances in our video data in which members utter the phrase “for example” in their constructions of turns-at-talk, we find that the temporal positioning of the phrase, in a retrospective-prospective manner (see Garfinkel, 1964: 229) with regard to the exemplifying component, is constitutive of producing the gestaltic sense of exemplification as a pair. Grounded in ethnomethodology and conversation analysis, the current study describes how members mobilize the formulative phrase for the organization of the gestalt, thus constituting exemplification as a social practice for two distinct social actions in the classroom: (1) accounting for one’s opinion and (2) confirming an understanding.

## Examples in the Literature

Despite the pervasiveness of exemplification as a social practice, it has largely escaped the attention of the social sciences as a topic in its own right, including in the field of ethnomethodology and conversation analysis. In this section, we first conceptually examine the notion of exemplification in order to later develop its conversation-analytic respecification (see Sormani, 2019). This involves introducing an ethnomethodological rendition of exemplification as a praxiological *gestalt,* highlighting that the work involved is to achieve the reflexive relationship between the exemplifying and the exemplified pair. We then move on to the next section, where we describe more empirical studies on the interactional make-up of exemplification, to which the current paper is situated as a contribution to the naturalistic study of exemplification-in-action.

### The Concept of Exemplification

In his introductory chapter on philosophy as a discipline, Sober (2021) uses the analogy of the “mammal” as an exemplified component to the exemplifying components, “human being,” “hippo,” and so on, to suggest that while he uses a variety of examples to describe philosophical work, the discipline is surely more than, and at least not equivalent to, the enumeration of those examples:

But giving examples doesn’t really answer the question of what philosophy is. If you asked, “What is a mammal?” and I showed you a human being, a hippo, and a cat, these examples might give you a *hint* about what a mammal is. However, citing examples isn’t the same as saying what it is to be a mammal.

(Sober, 2021: 3f., original emphasis)

In line with the epistemological tradition in logico-analytic philosophy, the quote above distinguishes the concept of exemplification from denotation (see Vermeulen et al., 2009), where the former merely *hints at* while the latter *says* what it is*.* Exemplification, in this field, is operationalized with the assumption that examples in themselves do not play a role in the making of meaning of the entity being exemplified. What one has instead is a given entity and its denotation as the meaning of that entity while exemplification is subjugated to concretizing, specifying or detailing that given meaning. Within this framework, it is neither exemplification, nor the setting in which it transpires, that constitutes the sense of the exemplified component, and the denotational meaning of the exemplified entity is what unilaterally affords disparate instances the label, “examples”. Other perspectives suggest a more transformative sense of meaning on the part of the exemplified component (e.g., Liu, 2017; Zillmann & Brosius, 2000). Zillmann and Brosius (2000), for instance, show how different forms of media can portray particular instances of our social life as examples, or prototypes, of a reality that may not actually exist in those terms. From this perspective, the meaning of the exemplified component, i.e., reality, is susceptible to change in terms of what is counted as example of that entity.^2^

Our position is that both frameworks above share the commonality that they do not approach exemplification as a phenomenon in everyday talk-in-interaction. That is to say, they are less concerned with describing the real-time sense-making work with which members in society establish for each other the intelligibility and meaningfulness of exemplification as it locally emerges as a relevant social practice in the course of an activity. In fact, we argue that accounting for exemplification as a social fact produced by and for members as they orient to it in their everyday life is antecedent to any serious conceptualization of exemplification in the social sciences. In the remainder of this text, we adopt an alternate approach to exemplification, inspired by ethnomethodology (Garfinkel, 1967; Heritage, 1984; Lynch, 1993). It describes and explicates the lived production of exemplification in its situated circumstances as members locally achieve its *gestaltic* character (see Eisenmann & Lynch, 2021; Hutchinson, 2022; Meyer, 2022) for all practical purposes.

Proposing that exemplification is an *achievement* of a gestalt between two components as a pair^3^ implies that there is work involved in the production of that pair. This work hinges upon the irremediable indexicality (Garfinkel, 2002; Garfinkel & Sacks, 1970) of exemplification, for the same things can be examples of one concept, and at other times, of another, and vice-versa. Simply put, everything in the world can be an example of something. This is to say that the exemplifying and the exemplified components do not carry a stable sense of reciprocal meanings. Instead, their relationship is an indexical one: they mutually give sense to each other. The ethnomethodological rendition as such is elaborated in Garfinkel’s “mis-reading” of Kuhn (1970) and the notion of “paradigms as shared examples”. What a scientific paradigm amounts to is members’ sharing of examples of scientific work in which certain instances are *discovered* (Garfinkel, 2022) to count as examples and others not, for the organization of their affairs as legitimate, official scientific work and in so doing being a competent member of that community:

One of the fundamental techniques by which the members of a group, whether an entire culture or a specialists’ sub-community within it, learn to see the same things when confronted with the same stimuli is by being shown examples of situations that their predecessors in the group have already learned to see as like each other and as different from other sorts of situations.

(Kuhn, 1970: 193f.)

If exemplification is, in fact, a discovery work in situ, what is to be discovered? The discovery to be made is the procedure, or the course of practical actions, of exemplification as a social practice. Put another way, it is not that exemplification as an a priori social fact informs researchers as well as members of the recognizability of its occurrence in interaction but that describing exemplification hinges upon its being accounted for as such by and for the members (and again also for the researchers) in the course of the practical tasks they are engaged in. As Garfinkel (2022) puts it, using the metaphor of a sketch map (i.e., exemplification as a formal-analytic phenomenon) and the actual journey of that map (i.e., exemplification as members’ phenomenon): “[I]t’s not that the sketch map is the thing we’re looking for. What we’re looking for is a sketch map used as part of the *journey* that it speaks of” (Garfinkel, 2002: 130; original emphasis). In our case, the “sketch map” would be the conceptual/logical link between the exemplifying and the exemplified components, while the “journey” would be the actual work of exemplification in situated occasions. The ethnomethodologically inspired perspective invites the question: What does exemplification as the work of its “journey”—its concerted production in real interactional time—consist of?

### The Work of Exemplification

While the work of exemplification can be found in all segments of social life, it may feature most commonly in the classroom and other educational settings where knowledge is constantly shared and modified. Early interest stems from the field of educational psychology on the facilitative role of examples for cognitive processing (e.g., Clark, 1971; Kellerman & Bialystok, 1997). In this line of research, “the analytic focus is placed on mapping out propositional relations between examples and target concepts” (Lee, 2004: 102). It is the target concepts (i.e., the exemplified component), and not examples themselves, that are the ultimate objects of learning, and therefore the focal phenomenon within this framework is how *instrumental* examples can be for the cognitive processing of those target concepts. Naturally, there is abundant research on the efficacy of exemplification as a pedagogical method in the field of (language) pedagogy (e.g., Byrd, 1998; Dong, 2013). In these two frameworks, the work of exemplification is not a *topic* of inquiry but a taken-for-granted *resource* (Garfinkel, 1967; Pollner & Zimmerman, 1970).

An alternative way to study the work of exemplification is to investigate it as a topic of inquiry in its own right (De Stefani et al., 2016; Gülich, 2003; Lee, 2004; Oliveira et al., 2020; Wortham, 1994). One of the earlier empirical works is Gülich (2003), who draws upon the principles of conversation analysis, analyzing exemplification as one of many conversational techniques used to mediate and transfer knowledge between medical experts and non-expert patients. Gülich (2003) emphasizes that “so-called ‘non-experts’ (in this case, patients) are also experts of a kind” (2003: 258), thus attesting to the fact that exemplification as a conversational technique is a collaborative achievement made by all parties to the interaction and not a more knowledgeable individual’s unilateral way of conveying knowledge.

Another study is De Stefani et al. (2016), which examines the work of exemplification as a resource for the multimodal phenomenon of note-taking, i.e., converting spoken discourse to written form. Employing conversation analytic methods, the study investigates note-taking practices in mutual-help groups and unpacks the “interactional history” of what is retrieved from the spoken group discussion as a case of an example to take note of in written form, or “recordables,” and how. Instead of treating exemplification as “discursive (syntactic, argumentative) properties in written texts, which are treated as static artifacts of human communication” (De Stefani et al., 2016: 112), the study shows that the social practice of exemplification is a dynamic and interactional achievement. The achievement therein is that exemplification preserves particularly relevant aspects of ongoing spoken discussions and thus constitutes meaning in its own terms.

Especially pertinent to our own research are studies on exemplification in the classroom. Drawing on ethnographic observations and recordings of English and History high school classes, Wortham (1994) explicates the forms and distributions of exemplification by coding instances of examples in his data along 18 dimensions, including variables such as activity structure (i.e., hierarchical/non-hierarchical), interactional function (e.g., interrogative, declarative) and interactional role (e.g., answering a question) (1994: 57–62). Statistical clusters among select variables mentioned above revealed that the membership of the speaker, i.e., teacher or student, played a marked role in different usages of examples. Findings include, for instance, that students are twice as likely to use explicit markers of exemplification, which include “for example,” than the teacher. While Wortham explains this in terms of students’ propensity for formal language, the study does not delve into the interactional design of the particular formulative phrase as it is constitutive of and contexted in specific actions. The current study addresses this gap by adjusting the focus from the social status of speakers to the sequential environment in which the formulative phrase is situated, showing how the phrase as a multimodal resource is deployed for the organization of social actions.

Based on data from English as a Second Language (ESL) speaking and composition classes, Lee (2004) analyzes instances of instructional examples used by the teacher in the course of talk-in-interaction. The analyses describe how the teacher positions examples as a timely response to students’ lack of understanding of a concept as demonstrated over multiple turns-at-talk. However, as the exemplified component, the target concept does not possess a static meaning. Rather, its sense is “progressively constructed in an array of interpretive moves by the teacher and her students” (Lee, 2004: 111), attesting to the locally emerging and reflexive relationship between the exemplified and exemplifying components. The study shows that the intelligibility of examples is not a corollary of the semiotic features *inherent* to the paired components but instead needs to be considered an ongoing *achievement* in and through constant sense-making work by participants to the talk as they locally and sequentially “demonstrate” (Sacks, 1992) for each other their assumptions and understandings for all practical purposes.

A more recent study is Oliveira et al. (2020), which looks at a teacher’s exemplification practices in a university biology class over the course of a semester. Identifying an array of factors (e.g., relatability of examples, extent of detail) involved in the effectiveness of the examples, the study describes how the teacher produces “memorable exemplification” as a way of instantiating learning experiences in the classroom. While both Lee (2004) and Oliveira et al. (2020) focus on the work of exemplification from the teacher’s side, describing a praxiology of pedagogy-in-action, the current study expands the naturalistic investigation of the work of examples in the classroom by analyzing exemplification practices from all members of the classroom community, both teacher and students, who are faced with a number of practical tasks, instructional matters being only one task among many. Moreover, the current study is an original attempt to apply the notion of gestalt to the phenomenon of exemplification, describing how the prospective-retrospective placement of the formulative phrase “for example” contributes to the intelligibility of exemplification as a social practice within the organization of particular social actions.

## Data and Methodology

The data for this study are collected from an advanced-level (i.e., C1^4^ and above) English language class that took place at a pedagogical institution affiliated with a public university in Switzerland, collected by one of the authors. Given that a central feature of examples is their “capacity of making abstractions comprehensible” (Zillmann, 1999: 86), they can be expected to be more frequent and prominent in the work of teaching and learning in the classroom. Exemplification could be even more prominent in a *language* classroom for the purpose of maintaining intersubjectivity between members of the classroom to accommodate issues of differing linguistic competence. This makes the language classroom an informative setting for this study, since it affords relevance, by and for the members, in revealing the “just how” of examples’ work in talk-in-interaction.

The language class examined in this study was held once a week (in a 75-min session) over the course of 12 weeks, amounting to eight courses in total. The participants of the class consisted of one American lecturer and seven adult students with different first language backgrounds. Each class was organized in a similar format, in which the lesson would begin with a 10-min speech by a different student every time on various topics of their choice, followed by a question-and-answer session, after which students would be grouped in threes and fours to discuss a podcast that they were assigned to every week. There were no restrictions as to the podcasts they could choose, but the teacher encouraged them to select ones that would promote lively discussion. The group discussion was allotted 45–50 min, taking up most of the class period, and the teacher in the meantime would go around the groups and join in their discussions, taking note of any linguistic errors and also facilitating more interaction. At the end, with 10 to 15 min remaining in the class time, the teacher would bring the class back together and go over some of the recurrent linguistic errors that they made during their group discussions. “For example” formulations were observed across all activity types mentioned above, but more instances were found during the group discussions, partly due to the majority of the class time being allotted to the activity.

The current study draws on the principles of ethnomethodology (Garfinkel, 1967, 2002, 2022; Livingston, 1987; Lynch, 1993) and conversation analysis (Sacks, 1992; Sacks et al., 1974; Schegloff, 2007). Recognizing everyday talk-in-interaction as “the primordial site of human sociality” (Schegloff, 1987: 102), ethnomethodology and conversation analysis aim to describe the methodical procedures of the constitution of social order from within. These procedures are constitutive of social phenomena that again reflexively constitute the intelligibility of the procedure as a procedure for the accomplishment of particular social actions. They are necessarily bound to the temporality of talk-in-interaction, composing a sequentiality of those procedures which, in turn, become the context for themselves (see ten Have, 2007, for an overview of the approach). Within this framework, our study is based on video-recordings of the target language classroom, using multiple visual and audio channels to capture both whole-class modes of teaching as well as group work and more localized interactions, amounting to approximately 11 h of recordings. The recordings were then transcribed using the Jeffersonian convention (Jefferson, 2004) for verbal talk-in-interaction, complemented with multimodal transcription for embodied conduct (Mondada, 2018), with the aim of preserving and representing the sequentiality of multimodal resources put into practice. A total of 50 instances of “for example” formulations were identified in this corpus, out of which the current study showcases six instances in which the formulative phrase is mobilized as an interactional resource for the organization of two distinct social actions: *accounting for one’s opinion* and *confirming an understanding*. The languages of the data are American English and English as a Lingua Franca.

## Analysis

Focusing on the interactional import of the formulative phrase “for example” (henceforth “FE”), we describe its temporal positioning in the course of talk, thus achieving two distinct social actions: (1) *accounting for one’s opinion* and (2) *confirming an understanding.* While these focal actions are not suggested as exhaustive for how FE constitutes social actions in talk-in-interaction, they were the most frequent and recurrent uses in our corpus and hence warrant particular investigation.^5^ Moreover, the two actions are informative for how exemplification can be distinctively delivered and made sense of for different kinds of actions; the first action, accounting for one’s opinion, has to do with the rhetorical dimension of exemplification to establish an argument, while the second action, confirming an understanding, is a device for maintaining intersubjectivity. As we will see in the analyses below, the prospective-retrospective placement of FE turns out to be consequential in organizing the praxiological gestalt of exemplification in and through each action.

### Accounting for One's Opinion

One of the more abundant sites of exemplification is argumentation and opinion-tellings, both within and outside the walls of the classroom. In this section, we analyze three excerpts to describe how the prospective placement of FE (when the exemplifying component is introduced after the phrase “for example,” i.e., FE-X) contributes to the action of accounting for one’s opinion during discursive activities in the classroom. We are using the phrase, “account for,” in the vernacular sense: the work of members in establishing the legitimacy, rationality, and validity of one’s opinion in so many words. Since students more often expressed their opinions of the podcast with each other, and the teacher was more often in the position of listening and taking notes, the cases of FE found for this study were nearly all from students.^6^

Excerpt 1 is a segment from a group discussion on a podcast about AIDS. As the excerpt begins, the teacher (TEA) prompts the students to give their opinions on whether someone can be held responsible for being sick. After the issue is briefly discussed between one student (STU1) and TEA, another student (STU2) volunteers an opinion in which he positions FE immediately after an explicit formulation of his stance on the matter (line 18).

*Excerpt 1 (BAS_20211209_21.03)* 
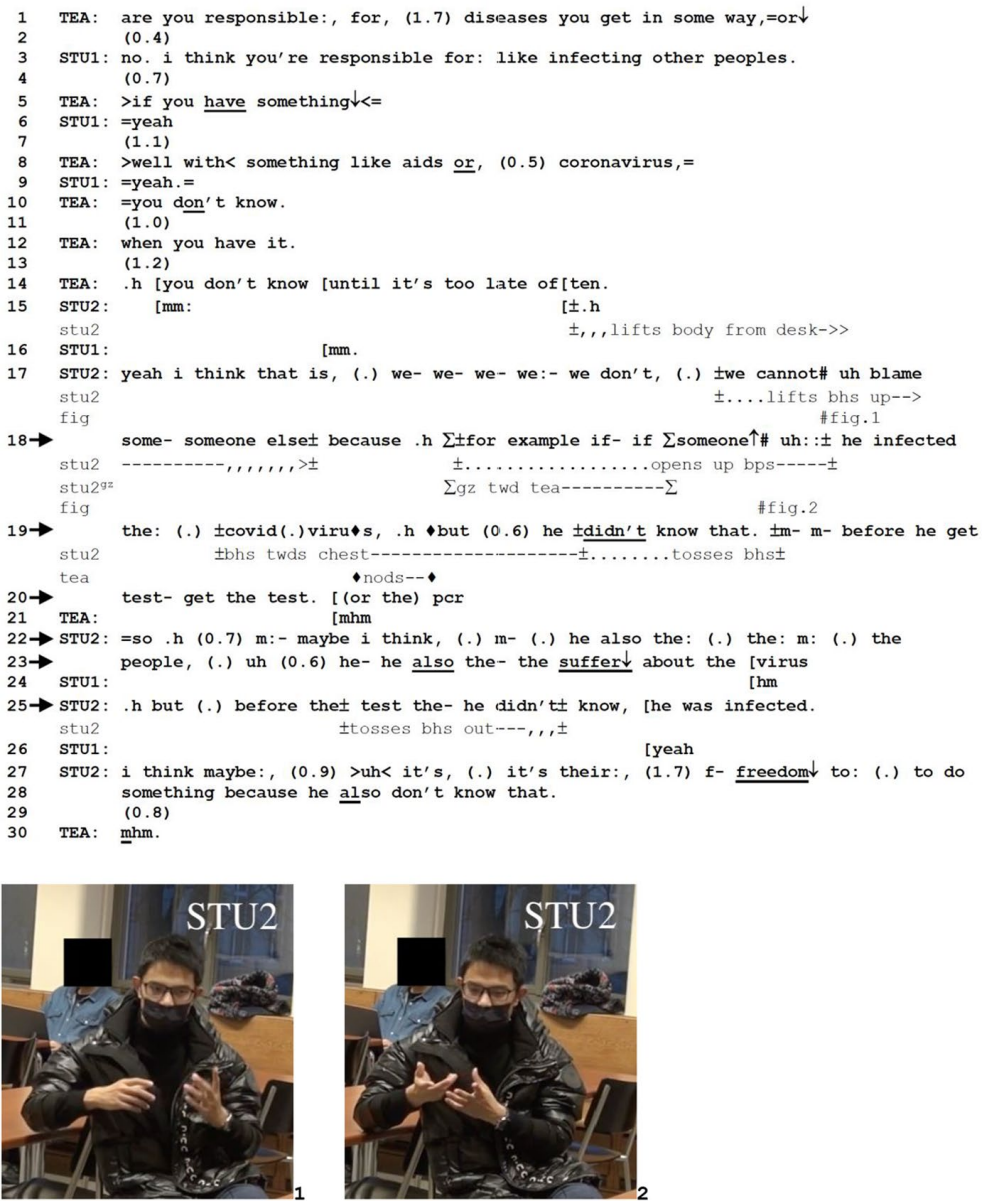


In lines 1–14, TEA and STU1 discuss how responsible a sick person is for their disease and STU1 acknowledges that there is responsibility for “infecting other people” (line 3). In lines 8 and 10, TEA points out that a person often may not know that they are infected “until it’s too late,” establishing a distinction between “knowing” and “being” (one can *be* something without *knowing* about it). While acknowledging STU1’s opinion, STU2 provides a counter opinion, suggesting that one should not “blame” (line 17) sick people even when they end up infecting others. The explicit display of his stance is accompanied by a lifting of his hands (Fig. 1), a similar gesticulation as has been observed for storytelling sequences in which tellers append gestures when an explicit formulation of their stance is relevant (Stivers, 2008).

STU2’s turn continues with the conjunctive “because,” and it is here that he produces the formulative phrase “for example” (line 18). The positioning of FE after “because” does the work of projecting that whatever example comes next has to do with a causal relationship between the previously stated opinion as the antecedent, or the exemplified, and reasonable grounds for this opinion, the exemplifier. FE is positioned prospectively, the exemplifying component coming right after the phrase. What comes after this prospective placement of FE is quite an elaborate example, spanning across an extended turn at talk (in this excerpt, lines 18–25). The exemplifying component is a hypothetical telling (Goodwin, 1990) with a conditional construction (double “if” in line 18) about a person who may have been infected by coronavirus without knowing that they had it until they had a positive test result. The example, therefore, marks a shift in concreteness from talking about virus infection generally to talking specifically about the coronavirus or “covid” (also tying to the teacher’s previous mention of coronavirus in line 8). The example-as-telling is designed in a chronologically organized series of events: getting infected (line 18) and not knowing it (line 19) until taking the test (lines 19–20), all the while suffering from coronavirus (lines 22–23) and still not knowing one has it (line 25).

The form of the exemplifying component as a telling is also coordinated by STU2’s gestures (as has been discussed for Fig. 1), and in this light, it is interesting to examine STU2’s deployment of multimodal resources at the point of the FE, including his gaze towards TEA and his raising of both his hands (Fig. 2). The multimodal configuration of FE as such seems to be an embodied account for what the exemplification is to be heard as: that is, as an account that supports his displayed stance. Note also the prosodic emphasis on the parts of the example that support his stance that sick people should not be blamed for infecting others (e.g., “he didn’t know” in line 19; “he also suffer” in line 23). During the production of the example, TEA produces nods (line 19) to the example-as-telling, akin to Stiver’s (2008) observations on recipients’ nods during tellings as displays of affiliation. The prospective placement of FE in Excerpt 1, therefore, does not merely project the insertion of an example in the middle of an opinion-telling—it also, and more importantly, prepares the recipient to listen for how the upcoming example supports, or accounts for, the opinion expressed.

Next, Excerpt 2 is from the same group discussion as Excerpt 1, taking place roughly 15 min later. At this point in the activity, the topic of this group’s discussion is coronavirus, rather than AIDS, which was the original theme of the podcast. Before the excerpt begins, STU2 has claimed that the notion of “freedom” has been distorted in the context of peoples’ conduct during the COVID-19 pandemic: simply irresponsible behavior is justified in the guise of freedom. Excerpt 2 begins as TEA follows up on this idea, enumerating concrete cases of such behavior, and asks STU2’s opinion on those. STU2 responds with an extended telling sequence during which he produces FE (line 16) to introduce specific cases of travel restrictions that he feels people should be subject to (line 17). In so doing, STU2 accounts for his opinion that freedom cannot be blindly respected in the face of a crisis.

*Excerpt 2 (BAS_20211209_38.11)* 
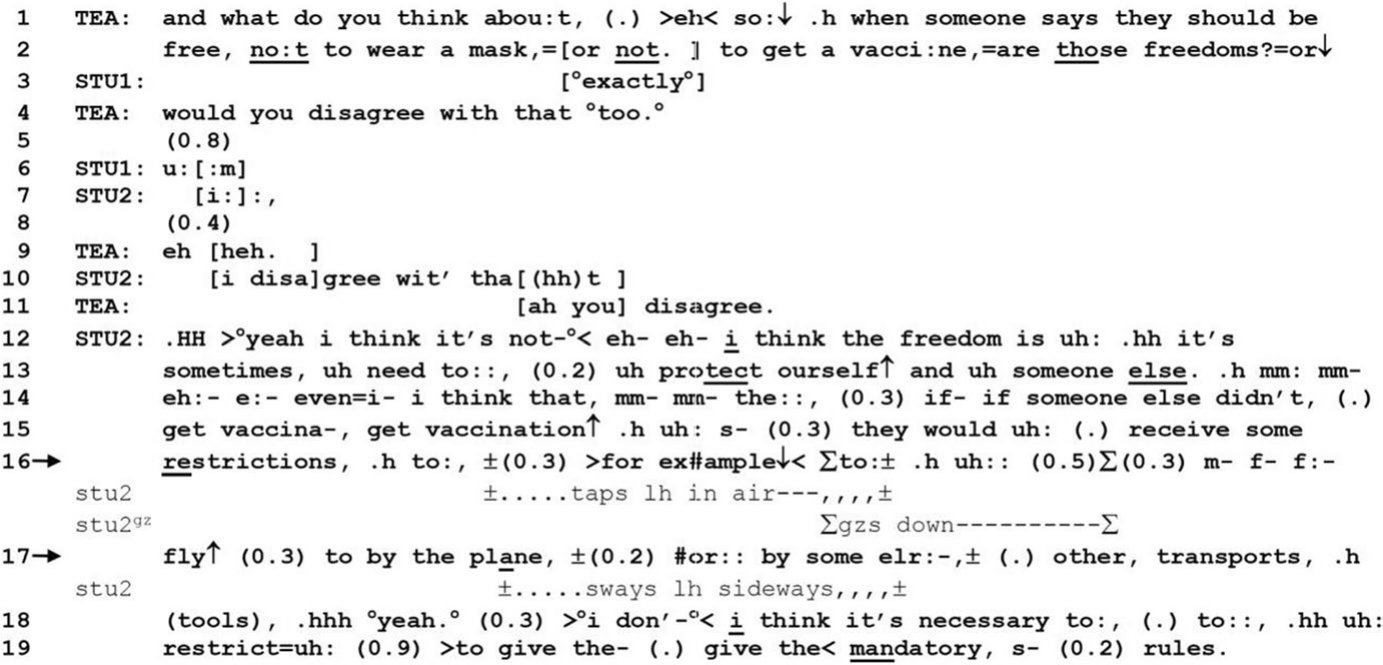


In lines 1–4, TEA posits a hypothetical case of someone arguing that they have the freedom to not wear a mask or get vaccinated and asks STU2 whether such behavior counts as a legitimate pursuit of freedom or not. While there is some projection and relevance on the part of STU1 to respond, STU2 begins his turn with an elongated first-person pronoun, “I” (line 7), treating TEA’s turn as directed to eliciting his own personal opinion. After a slight pause, STU2 restarts his turn with an explicit display of his stance, saying, “I disagree with that” (line 10). The laughter particles embedded in this turn may be due to the lack of any mitigation in displaying his stance, a bluntness that is reified as STU2’s response recycles TEA’s question (“would you disagree with that too,” “I disagree with that”). At the same time, the laughter particles may be mitigating his disagreement, which is ironically an agreement with TEA’s polar structure of the question.

While TEA is early to respond (line 11) to STU2’s turn, possibly treating line 10 as a complete turn constructional unit (TCU), STU2 goes on to produce a multi-unit turn starting in line 12. Given that this multi-unit turn is positioned after an explicit display of his opinion, we can expect it to be some elaboration of his opinion. Indeed, in lines 12–13, STU2 begins the turn with an “I think” (line 12) and repeats the keyword “freedom” to argue that the construct should be used to “protect ourself and someone else” (line 13). After some self-initiations of repair, STU2 again begins with “I think that” (line 14) and recycles TEA’s hypothetical case of someone not wanting to be vaccinated to make the case that such people should “receive some restrictions” (lines 15–16). While the turn is appended with a projected complement *to X*, this is cut off while STU2 gesticulates, tapping his left hand in the air, holding onto the conversational floor.

To characterize the talk up to now, STU2 has begun with a more general formulation of his stance on freedom and its meaning (lines 12–13), and then moved on to what that means in a specific case (lines 14–16). This makes recognizable for the recipient, in real time, a discursive structure of an argument and is also the sequential environment in which FE gets produced (line 16). The FE is prospectively positioned, the exemplified being the antecedent, “restrictions,” and the exemplifier, restrictions in a specific area of everyday life, namely, transportation (line 17). Unlike Excerpt 1, the exemplifying components here are not hypothetical but evidential: at this point in the pandemic, people who were not vaccinated were indeed, in reality, subject to restrictions, one of the more prominent ones being air travel and other forms of transportation. The conspicuousness of the example, therefore, makes it difficult to listen to it for its informativeness or explicativeness.^7^ Instead, STU2 accounts for his opinion by producing an FE that projects a showcasing of real-life examples of restrictions, thus rhetorically strengthening and empirically validating his point.

In the final excerpt, TEA and students are discussing a podcast on the Taliban’s takeover of Afghanistan. Prior to the excerpt, one of the students (STU1) shared his thoughts on the podcast being potentially skewed in its representation of the situation for the sake of soliciting empathic responses from its audience. The excerpt begins with TEA agreeing with STU1. In response to TEA’s alignment with him, STU1 elaborates on an alternative approach to the situation, producing FE (line 63) to introduce an example of a conference that took place in Brussels to account for his opinion.

*Excerpt 3 (BAS_20211118_01.17.34)* 
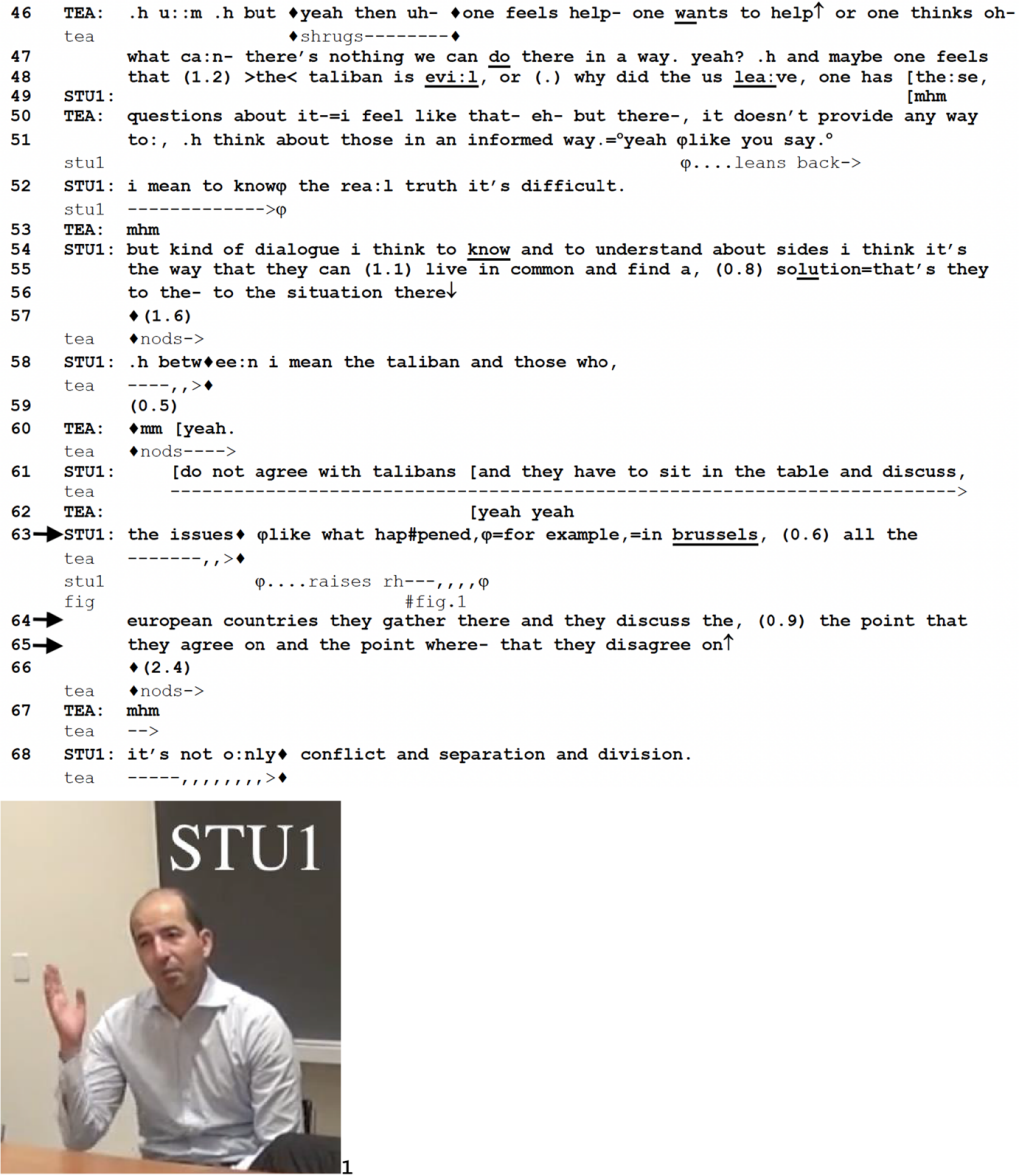


In lines 46–51, TEA talks about how the rhetorical purpose of the podcast, namely to invoke emphatic reactions from the audience about the situation of women in Afghanistan, may lead to morally laden reactions in the audience (i.e., “the taliban is evil or why did the US leave” in line 48) and, as a consequence, miss out on promoting an “informed way” (line 51) for the audience to approach the situation. TEA attributes this stance to STU1 using a latched-on increment (“like you say” in line 51). The orientation and attribution of such a stance to STU1 in this sequential environment, that is, a transition relevance place (TRP), gives STU1 space to further his own stance, whether in the form of elaborating on TEA’s remarks or perhaps even refuting them. The space as such is indeed recognized by STU1, who adjusts his body, a gesture that he initiates upon TEA’s explicit orientation to him and maintains as he begins the next turn-at-talk (lines 51–52).

STU1 begins his turn in line 52 with “I mean,” which—unlike some previous findings in similar settings—is not used here to “correct previous statements and dysfluencies” (Xue & Lei, 2016: 297), but projectively as a first part of a contrastive conjunction that is introduced by the use of “but” in line 54. His response first acknowledges TEA’s formulation of his stance (line 52), that is, the podcast has a difficult time providing an accurate representation of the situation, but after the contrastive conjunction, STU1 shifts the topic to how the Taliban’s occupation of Afghanistan could be dealt with in practical terms. Here, he proposes that the crucial thing is to find a consensus between the two opposing parties, the Taliban and the people who are against them (lines 54–63). It is at this point, having elaborated on his stance on a practical solution, that STU1 projects an exemplification, saying, “like what happened” (line 63). Similarly to the previous two excerpts, STU1 gesticulates, in this case, raising his right hand (Fig. 1) at this specific juncture. Unlike the previous two excerpts, however, STU1 prefaces the FE with yet another conventional token of exemplification, i.e., the adverbial “like”. He goes on to still append FE, after which the exemplifying component of the Brussels case is introduced. Both semantically and syntactically speaking, FE may not seem necessary for the design of this turn. We argue, however, that the *formulative* aspect of FE becomes clearer in such seemingly redundant cases, because its work evidently lies not just in flagging an example, but more importantly, in *labeling* something as an example. The labeling, or formulating, of certain things as examples, using such phrases as FE, in the midst of displaying a stance seems to be part of what it means to *account for,* rather than just offer, an opinion.

The FE is placed prospectively, the exemplifying component that follows being a stand-alone phrase “in brussels” (line 63). Brussels, a location geographically distant from Afghanistan, is suggested by STU1 to be sufficient as a lexical item to refer to a moment when the systematic seeking of consensus took place among European countries. STU1 is thus providing a model exemplar, the Brussels case, for moving beyond “conflict and separation and division” (line 68) in Afghanistan, accounting for the opinion he had expressed in lines 54 to 63. After the prospective positioning of FE, there is a 0.6-s pause (line 63). During this pause, before the same speaker starts producing the next TCU (“all the european countries...” in lines 63–64), “for example in brussels” has the features of a pivot (see Norén & Linell, 2013): it could be heard as incrementing the previous TCU (“... what happened, for example in brussels”), as well as starting a new TCU (“for example in brussels,...”). In the sequential production of STU1’s turn at talk, the pause can be heard either as marking a TRP or as a pause at the point of maximum grammatical control (Schegloff, 1996). Despite this potential ambiguity, it seems that participants treat the pause unambiguously as the latter—none of them launches a new turn at talk during the pause or displays preparation for it. Similarly to the previous two excerpts, the prospective positioning of FE in Excerpt 3 reflexively composes STU1’s ongoing turn as an opinion-telling and also constitutes the impending TCUs as accounting for that opinion in the specific form of exemplification. Put another way, the prospective placement of FE is a device that prepares recipients to listen for whatever comes as an exemplifying component—in this case, Brussels—as an account for, or claiming of, STU1’s opinion.

Analyses of the three excerpts have shown how the prospective positioning of FE (FE-X) constitutes the practice of exemplification, thus making recognizable the action of accounting for one’s opinion. The exemplifying components were introduced as materials to support a previously stated view of the speaker, whether by constructing a hypothetical narrative (Excerpt 1), enumerating specific entities (Excerpt 2), or offering an actual model exemplar (Excerpt 3). In terms of turn design, FE might occur as part of a causal relationship (“because” in Excerpt 1), an attachment to a complement of a previous predicate (“to” in Excerpt 2), or in a pivot-like construction (Excerpt 3). We have described how the prospective placement of FE after an explicit formulation of one’s stance can be understood in reference to the organization of a story preface (see Sacks, 1972). The story preface is routinely situated to hint at the teller’s stance before the actual body of the telling, thus preparing recipients to listen for the teller’s stance. When FE is placed immediately after an explicit formulation of one’s stance, it is heard as prospectively introducing material that works to account for that stance, where “the sense of the prior unit [FE] as a ‘pre-’ becomes obvious in retrospect” (Streeck & Jordan, 2009: 94). On the part of the recipients, hearing FE in this sequential environment then means listening not just for what the exemplifying component is, but for how the example accounts for the stance just provided. The work of achieving a gestalt of exemplification, therefore, lies not only in identifying the pair but also in identifying the pair in its temporal and practical situatedness, in this case, how FE furnishes the prospect of not just an example but the example-as-an-account.

### Confirming an Understanding

In addition to situations where opinions are accounted for, examples are routinely formulated in sequential environments where participants convey knowledge to each other. Exemplification as a practice allows speakers to demonstrate their understanding (Sacks, 1992, Vol. II: 140–142) of knowledge and confirm to each other that such understanding is correct for all practical purposes. In a language classroom, such knowledge might be related to vocabulary, grammar, pragmatic expressions and other linguistic structures, as well as cultural knowledge. In this subsection, we demonstrate how the action of confirming an understanding is constituted by the practice of exemplification that takes the form of a retrospective FE (i.e., X-FE). Exemplification can demonstrate one’s understanding when it remains to be confirmed by the recipient (Excerpt 4) but also to pursue confirmation of the other’s understanding (Excerpts 5 and 6).

Excerpt 4 is taken from a longer sequence of talk in which TEA and students are clarifying and discussing the notion of an “intellectual” with regard to the structure of employment in modern societies. The group is discussing this topic in reference to a podcast they have listened to on public intellectuals, which raises the question of what kinds of people actually count as “public intellectuals”. At the beginning of Excerpt 4, TEA continues to elaborate on the definition of an intellectual with some exemplary cases, after which one of the students (STU) offers an example of an intellectual with retrospective FE (line 26) as a way of confirming her understanding of TEA’s explanation.

*Excerpt 4 (BAS_20211111_38.31)* 
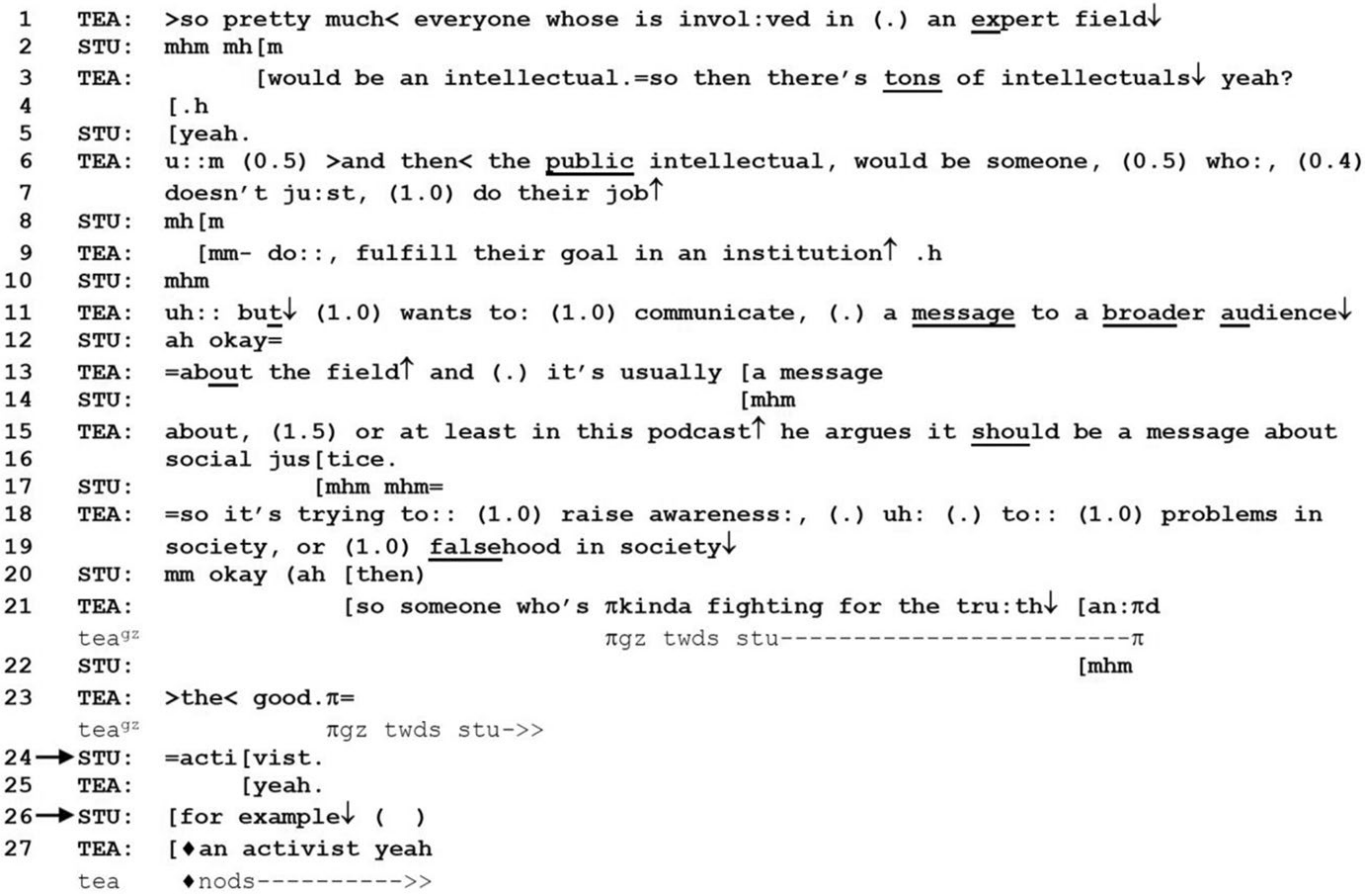


Lines 1–3 conclude TEA’s explanation of the word intellectual, with which STU aligns using continuers (lines 2 and 5). In line 6, TEA introduces a more specific type of an intellectual, a “public intellectual,” explaining it in a relatively long sequence in lines 6–23, initially in contrast to other intellectuals (“doesn’t just do their job, fulfill their goal in an institution”), and from line 11, providing a positive definition of how public intellectuals push for more societal contribution. In line 24, STU demonstrates her understanding of the teacher’s explanation by providing an example of a public intellectual: “activist”. In line 26, FE is used retrospectively as an increment. “Activist” is thus recast as a representative member of the category of public intellectuals rather than a synonym for the expression. In so doing, STU formulates her previous TCU as an example that demonstrates an understanding of the exemplified component rather than an alternative proposal that could replace the exemplified component. Prosodically, FE is hearable as an increment and itself is designed as a turn-concluding item with falling intonation. In overlap with STU, TEA repeats the given example (line 27), confirming STU’s understanding of his explanation, while also providing an embedded correction (Jefferson, 1987), adding the indefinite article “an”. TEA then explicitly accepts the word “activist” as indeed being a correct example of a public intellectual. By providing the example, and retrospectively marking it as such by the use of FE, participants are able to collaboratively demonstrate and confirm STU’s understanding of TEA’s explanation of what a public intellectual may be.

In Excerpt 5, TEA and students have been discussing the legality of surrogate pregnancy in different countries. Prior to the excerpt, one of the students (STU2) asked whether surrogate mothers are paid for the task in countries that have legalized the procedure. The excerpt begins with TEA responding to STU2’s question, at which point another student (STU1) elaborates on TEA’s response with an example (lines 12–13) appended with a retrospective FE (line 15). The example, in this way, becomes an opportunity for STU1 to pursue STU2’s understanding of TEA’s response.

*Excerpt 5 (BAS_20211209_01.03.07)* 
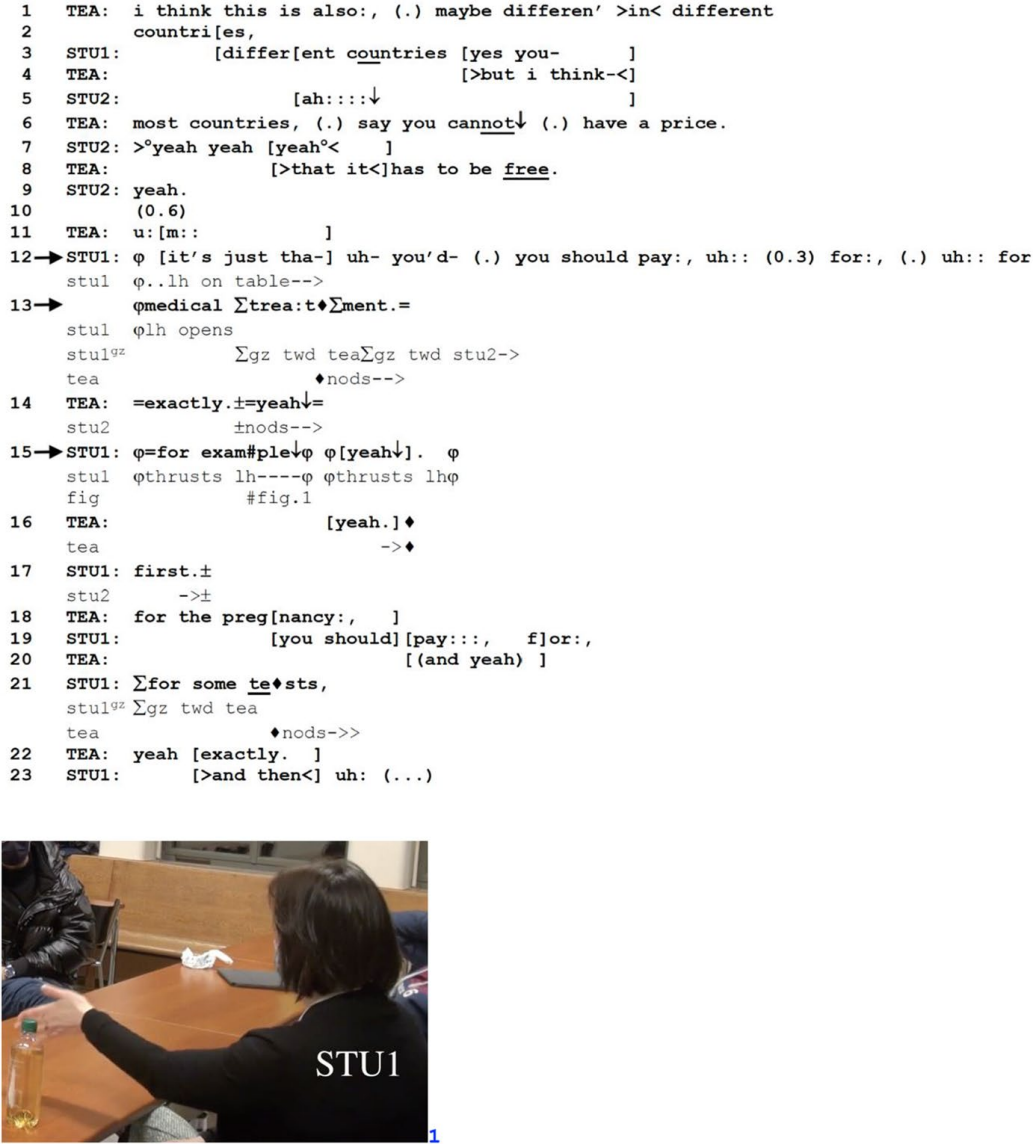


In his utterance in lines 1–8, which consists of multiple TCUs, TEA suggests that in “most countries,” surrogacy has to be done free of charge. STU2 claims understanding through acknowledgement, change-of-state and agreement tokens (lines 5, 7 and 9), in overlap with TEA’s talk. After a 0.6-s silence in line 10, TEA produces a prolonged trouble marker “u:m::” which may be a response to the lack of further uptake by STU2. At this point, STU1 self-selects to suggest an example of an exception, namely, necessary “medical treatment” (line 13), where payment is required. In so doing, STU1 demonstrates her understanding of what TEA means by saying that surrogacy is supposed to be “free”—the service itself should not be monetized but necessary medical costs for the surrogate mother should still be paid for. TEA agrees in line 14 (“exactly”), upon which STU1 latches FE,^8^ followed by a turn-final token “yeah” with a falling intonation contour. Like in Excerpt 4, FE here features as a standalone TCU, highlighted by STU1’s left-hand open-palm “offering” gesture (Streeck, 2009: 184) (Fig. 1). After the occurrence of FE, in line 16, TEA proffers yet another “yeah” similar to that in line 14, and in exact overlap with the STU1's “yeah” in line 13. TEA further explicitly confirms STU1’s understanding with an increment (“for the pregnancy” in line 18) that specifies the sense of STU1’s “medical treatment”. STU1 in turn continues elaborating on her example (lines 19–21), followed by TEA's agreement in line 22 recycling “exactly”.

In Excerpt 5, FE is positioned retrospectively to formulate “medical treatment” as an example of an exception where payment is necessary despite surrogacy in itself being “free”. The recipient’s repeated use of “yeah” (lines 14 and 16), immediately before and after FE, indicates that FE retrospectively formulates “medical treatment” as being illustrative of a larger set of objects that are not “free”. The recognizability of this as unambiguously an *example* makes conditionally relevant in the next turn the acknowledgment of an exemplification in practice (“yeah” in line 16). The status of “medical treatment” as an example is further elaborated by STU1, who in line 17 recasts this exemplifying component as a first item of a sequentially ordered set or a list (“first” followed by “then” in line 23). The close relationship between listing and exemplifying has already been noted in previous studies (see De Stefani et al., 2016).

Unlike Excerpts 4 and 5 above, in the next excerpt, it is the teacher who offers an example to pursue confirmation of students’ understanding of his own instruction. In Excerpt 6, TEA projects on a large screen a list of errors that students made during their group discussions that he took note of and explains why they are incorrect and how they can be fixed. TEA points out a grammatical error, the lack of an article after the determiner “such,” for which one of the students (STU) raises the possibility of an alternative rule. TEA responds with an extended instruction on the grammatical rule, closing it by proffering an example (line 21) to confirm the students’ understanding of his explanation.

*Excerpt 6 (BAS_20211104_01.34.03)* 
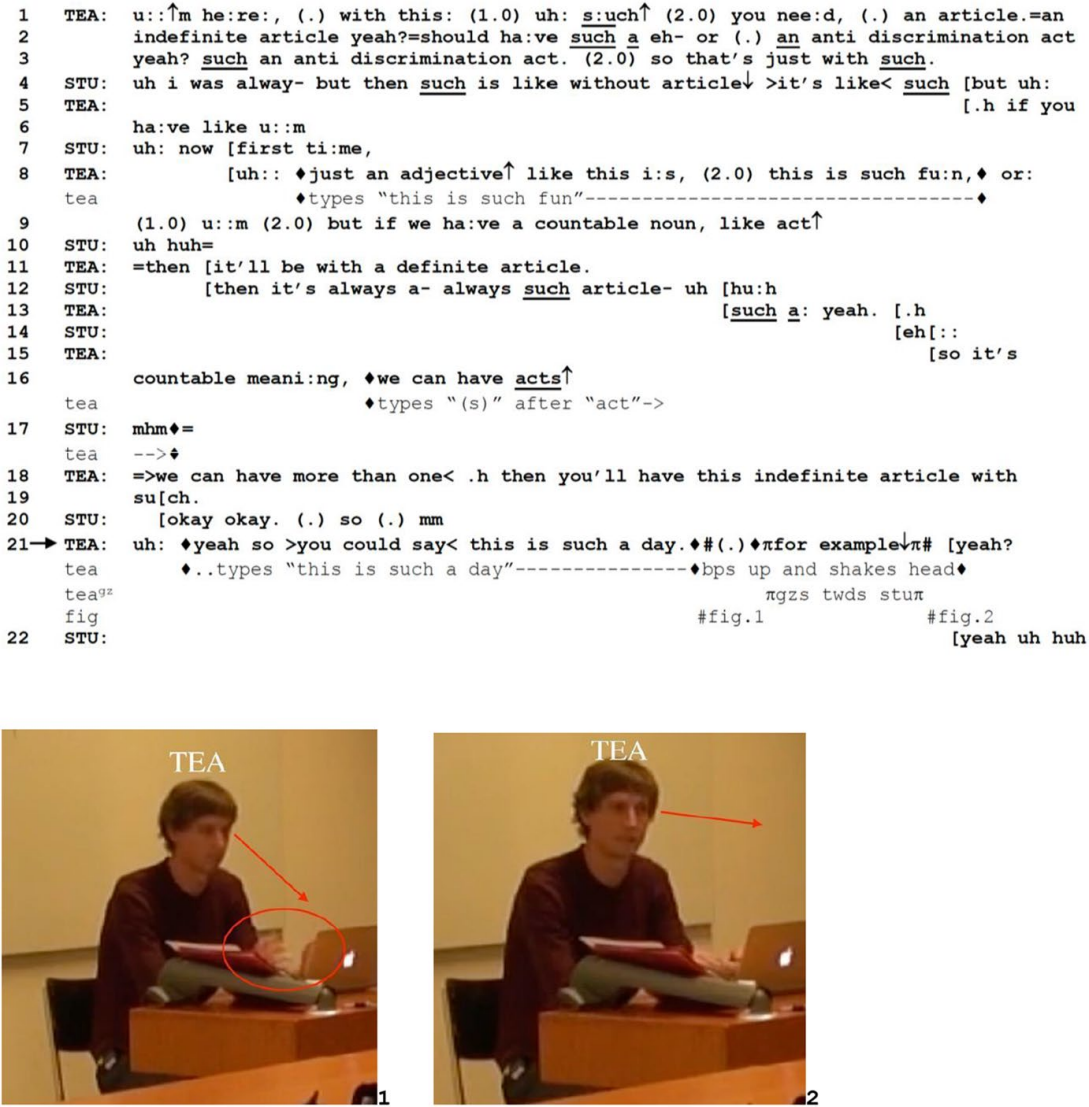


The long pauses and hesitation markers in line 1 index TEA’s parallel work on the laptop, such as highlighting and selecting elements of the text and typing the correct version, a process publicly visible to all students as his laptop is projected onto the larger screen. After STU’s questioning of the correct rule in line 4, TEA explains the general rule of using indefinite articles after “such” (lines 5–11). In line 12, STU demonstrates her understanding of the rule by finishing TEA’s sentence in overlap, and the explanation sequence seems to be closed in line 20 by STU’s “okay okay” (see Betz & Deppermann, 2021), produced with falling intonation. However, after a micropause, she reopens the sequence by adding “so mm,” which can be heard as pointing to trouble. TEA treats this as a request for an example, and after tying with “so” provides one in line 21, prefacing it with a conditional “you could say,” which already prefaces that what comes next may be an example. Thereafter comes the example, “this is such a day,” during which TEA looks at the laptop screen (Fig. 1) and types the phrase on the laptop so that it is publicly visible. It is at this point that TEA utters retrospective FE with falling intonation. This is accompanied by TEA looking up from the screen and gazing towards STU (Fig. 2). In this way, the retrospective FE is hearable not only as opening a TRP but also as furnishing the conditional relevance for STU to confirm her understanding. TEA himself orients to this hearing by appending a tag question (“yeah?” in line 21). In turn, STU provides acknowledging tokens “yeah uh huh” (line 22) in partial overlap, claiming understanding of the explanation.

To summarize, we have shown in this subsection that speakers use FE to confirm their understanding of a previous explanation given by another person (Excerpt 4) or to confirm the other person’s understanding of an explanation given by themselves (Excerpts 5 and 6). The phrase demonstrates an understanding and offers an instance of a membership category (“activist” as a type of “public intellectual”), an exception (“medical treatment” as something that is not for “free”), or a grammatical rule (“this is such a day” as instantiating the rule “countable nouns after *such* need an article”). The form of exemplification described here is more succinct, consisting of relatively short exemplary phrases, followed by a confirmatory response in the next turn. Through the sequential positioning of the exemplified-exemplifying pair, whatever comes after the explanation of the exemplified component is heard as actualizing, realizing, or instantiating the conversational object. That is to say, the exemplified component attains the feature of being applicable and the exemplifying component attains the feature of being applied. The retrospective FE, then, is a member’s real-time account of the locally emerging gestalt of exemplification.

## Discussion

Semantically, the phrase “for example” (FE) seems to mark a shift in concreteness, or establishing a relationship between two areas of knowledge with a distinct level of abstraction, dealing with “processes of generalization and specification” (Bilmes, 2020: 52). This perspective, however, does not account for the various ways FE is actually used in sequences of classroom interaction. In our paper, focusing on the temporal positioning of FE for the practice of exemplification within the actions it achieves, we argue that FE is a device for recipient design, used prospectively or retrospectively, as a formulative phrase (Garfinkel & Sacks, 1970)—prospectively in the sense of “what you are going to hear next is an example,” and retrospectively, “what you just heard was an example”.

We have shown that the formulative phrase FE can be used to *account for one’s opinion* and to *confirm an understanding*, i.e., to accomplish two distinct social actions. First, the prospective placement of FE is akin to a story-preface for the action of accounting for one’s opinion, where FE is heard to introduce some supporting material for one’s opinion. The fact that FE can be heard as prospective to not just an example, but an account, shows that members in argumentation “attend to a visible, consequential future that they attempt to structure in specific ways” (Goodwin, 2006: 459). The intelligibility, legitimacy, rationality and rigor of exemplification is inseparably grafted onto the progressivity of the argumentation being made—FE does not project only an example, but also good reasons for an opinion. The work of the exemplification is therefore part and parcel of the work of argumentation, in this case, accounting for one’s opinion. Secondly, our analysis shows that examples can also be formulated explicitly in retrospect, which is related to the work of confirming an understanding. FE is produced incrementally after an exemplifying component that is relatively brief and succinct. The retrospective formulation of the preceding TCU as an example contributes to the conditional relevance of a confirmation of understanding in the next turn. This social action is closely related to the maintenance of intersubjectivity and the establishment of shared knowledge that provides material to be elaborated upon in subsequent talk. Furthermore, we have shown the varied forms that exemplification takes in the organization of these two actions: elaborate narrative constructions (Excerpts 1 and 5), single terms or phrases (Excerpts 2, 3 and 4) and specimen performances (Excerpt 6).

For both actions, the work of exemplification rests in achieving the gestalt of the exemplified–exemplifying pair. The gestalt of exemplification is constituted through the sequential order of talk, with FE being oriented either prospectively or retrospectively. Our analyses reveal that the temporal positioning of the phrase in reference to the exemplifying component, whether prospective or retrospective, is consequential for the intelligibility of the exemplified–exemplifying pair and thus for the organization of the two focal actions. Exemplification is a praxiological gestalt (Hutchinson, 2022), its recognizability and consequentiality being achieved in the real time of social interaction (Au-Yeung & Fitzgerald, 2022). The work of exemplification is therefore intertwined with the lived production of social order for all practical purposes. Rather than focusing on the intelligibility of a pair—the exemplifying and the exemplified—as a perceivable gestalt in itself, we have examined how it is made intelligible-in-action and incorporated into the temporal structures of ordinary activities.

The work of exemplification studied in this article was accomplished by the participants as part of classroom practice. Not only are the actions explicated in the previous section tied to classroom activities, but these actions also reflexively constitute the classroom as a particular institutional setting. Indeed, from the perspective of ethnomethodology and conversation analysis, this is true of all social actions. Despite the fact that our empirical materials come from a single setting, we propose our findings as potentially relevant outside the educational realm: to what extent our analytical account of the work of giving examples offers “observations *recognizable* in other empirical settings” (Ziewitz, 2017: 12, original emphasis) remains to be specified by further research. Other directions for future studies of the work of examples in social interaction include an inquiry into other forms of exemplification. While examples marked explicitly with FE are common, there are many other constellations of resources for exemplification, which may be investigated in further research. Furthermore, instances of members’ orientations to the work of giving examples—suitable examples, confusing examples and so on—would be especially illuminating, providing opportunities to explore the sense-making practices related to this ubiquitous phenomenon in everyday and professional talk.

## Conclusion

Everyone is familiar with examples—we know how to give, request and recognize them in various scenes of everyday life. We know how to distinguish good examples from bad ones. We know that giving an apt example makes a difference and that examples can “lighten the atmosphere” (Garfinkel, [1948]^2006^: 153) during explanations of heavy, intellectually impenetrable subjects. Taking such “grossly apparent facts” (Sacks et al., 1974) about examples as a point of departure, this paper aimed to provide an exploration of members’ work of giving examples as a methodic procedure for the organization of social actions as they engage in their practical affairs, hence constituting their “*bricolage* expertise” (Garfinkel, 2022: 136). An example may be given by a speaker, but correctly figuring out the exemplified domain constitutes part of the interlocutors’ local interactional competence (Psathas, 1990). While an example, and what counts as one, can be defined in so many words, what its work consists of in the course of social interaction remains to be continuously attended to and made sense of by the members. The omnipresent tacit question in the work of exemplification is—as posed by Bateson’s student cited above in the introduction—“An example of what?”.

The aim of this study was to investigate exemplification as practical interactional work by focusing on the sequential organization of talk-in-interaction, analyzing the occurrence of the formulative phrase “for example” in the perspicuous setting of the language classroom. We have addressed the prospective-retrospective positioning of the focal phrase and considered its relationship to the distinctive actions achieved by exemplification as a social practice. On the one hand, the prospective positioning (FE-X) formulates the upcoming talk as an example, establishing a pair with the antecedent exemplified component in real time. On the other hand, the retrospective positioning (X-FE) reformulates the previous utterance as an example, ensuring the recognizability of a pair within the broader sequential environment of the talk. In addition to highlighting the interactional work of exemplification, this study also outlined avenues for future research, thus reporting on an exploration of just one out of many directions that naturalistic studies of exemplification can take.

## Appendix

### Multimodal Transcript Conventions (Short Version)

Embodied actions are transcribed according to the following conventions developed by Lorenza Mondada (see Mondada, 2018 for a conceptual discussion). https://www.lorenzamondada.net/multimodal-transcription

**: Descriptions of embodied actions are delimited between
++: two identical symbols (one symbol per participant and per type of action)
∆∆: that are synchronized with correspondent stretches of talk or time indications.
*--->: The action described continues across subsequent lines
--->*: until the same symbol is reached.
>>: The action described begins before the excerpt’s beginning.
--->>: The action described continues after the excerpt’s end.
…..: Action’s preparation.
----: Action’s apex is reached and maintained.
,,,,,: Action’s retraction.
ric: Participant doing the embodied action is identified in small caps in the margin.
fig: The exact moment at which a screenshot has been taken
#: is indicated with a sign (#) showing its position within the turn/a time measure.

**Abbreviations used in transcript**

bhs: Both hands
bps: Both palms
lh: Left hand
rh: Right hand
gz: Gaze
twd: Towards

## Footnotes

1 *Exemplification* is used in this article to gloss what people, in vernacular talk, account for as “giving examples,” “thinking of examples,” “offering examples,” “coming up with an example” and so on.

2 See also Sacks (1992) on “exemplary occurrences” as “a single case [where] the rule might get changed” (1992: 197). The idea of a rule here is that even if there are instances in society that run contrary to what is expected of the normal state of affairs, the rule as that very normative expectation would still stand and not be modified and those instances would be singled out as exceptions. Sacks points out, however, that single occurrences can be made sense of as exemplary occurrences, in which case members’ knowledge of how things should be could altogether shift.

3 The idea of perceiving two entities as a unified *pair* comes from Gurwitsch (1964), who works out a phenomenological discussion of how two dots are available to the perceiver as more than the enumeration of one and another, but already at the outset as a pair, constituting their sense by implicating each other. However, see Eisenmann and Lynch (2021) for a discussion on how an ethnomethodological approach respecifies such a perception as an ongoing praxiological achievement, and hence is ultimately post-phenomenological (Hutchinson, 2022).

4 Within the Common European Framework of Reference for Languages (CEFR), C1 is the second highest level of linguistic proficiency of the six levels in total. According to CEFR, a C1 level suggests that the person is a proficient user who is able to communicate in the target language for more professional activities, in addition to everyday communication.

5 Although, in some instances, the use of FE might achieve more than one of these actions, the excerpts presented and analyzed in this paper seem unequivocal.

6 The few instances of FE produced by the teacher for this action were when the teacher participated as a student, so to speak, when he joined in the group discussions for various practical reasons, including instances where there was a smaller number of students in a certain group or when he would try to fill in long pauses during discussions by proffering an opinion.

7 At the height of the pandemic, the fact that people were restricted from using public transportation depending on vaccination status and mask-wearing was well-established and shared, and therefore conspicuous, knowledge.

8 The occurrence of “example” might also be touched off by “exactly” in the preceding turn, in the sense of a “sound-row” (Jefferson, 1996), the two words being “utterances with more or less extensive internal sound repetition” (Sacks, 1968: 7).
